# Molecular image–guided surgery in gynaecological cancer: where do we stand?

**DOI:** 10.1007/s00259-024-06604-1

**Published:** 2024-01-18

**Authors:** Giusi Pisano, Thomas Wendler, Renato A. Valdés Olmos, Giorgia Garganese, Daphne D. D. Rietbergen, Francesco Giammarile, Sergi Vidal-Sicart, Maaike H. M. Oonk, Michael Frumovitz, Nadeem R. Abu-Rustum, Giovanni Scambia, Vittoria Rufini, Angela Collarino

**Affiliations:** 1 Section of Nuclear Medicine, University Department of Radiological Sciences and Haematology, Università Cattolica del Sacro Cuore, Rome, Italy; 2 Department of Diagnostic and Interventional Radiology and Neuroradiology, University Hospital Augsburg, Augsburg, Germany; 3 Chair for Computer-Aided Medical Procedures and Augmented Reality, Technical University of Munich, Garching, Near Munich Germany; 4 Interventional Molecular Imaging Laboratory & Section Nuclear Medicine, Department of Radiology, Leiden University Medical Center, Leiden, The Netherlands; 5 Gynecologic Oncology Unit, Department of Women, Children and Public Health Sciences, Fondazione Policlinico Universitario A. Gemelli IRCCS, Rome, Italy; 6 Section of Obstetrics and Gynecology, University Department of Life Sciences and Public Health, Università Cattolica del Sacro Cuore, Rome, Italy; 7 Nuclear Medicine and Diagnostic Imaging Section, Division of Human Health, International Atomic Energy Agency, Vienna, Austria; 8 Nuclear Medicine Department, Hospital Clinic Barcelona, Universitat de Barcelona, Institut d’Investigacions Biomèdiques August Pi iSunyer (IDIBAPS), Barcelona, Spain; 9 Department of Obstetrics and Gynaecology, University Medical Center Groningen, University of Groningen, Groningen, The Netherlands; 10 Department of Gynecologic Oncology and Reproductive Medicine, The University of Texas MD Anderson Cancer Center, Houston, TX USA; 11 Gynecology Service, Department of Surgery, Memorial Sloan Kettering Cancer Center, New York, NY USA; 12 Nuclear Medicine Unit, Fondazione Policlinico Universitario A. Gemelli IRCCS, Rome, Italy

**Keywords:** Image-guided surgery, Gynaecological cancers, Sentinel node biopsy, Hybrid tracer, Robotic surgery

## Abstract

**Purpose:**

The aim of this review is to give an overview of the current status of molecular image–guided surgery in gynaecological malignancies, from both clinical and technological points of view.

**Methods:**

A narrative approach was taken to describe the relevant literature, focusing on clinical applications of molecular image–guided surgery in gynaecology, preoperative imaging as surgical roadmap, and intraoperative devices.

**Results:**

The most common clinical application in gynaecology is sentinel node biopsy (SNB). Other promising approaches are receptor-target modalities and occult lesion localisation. Preoperative SPECT/CT and PET/CT permit a roadmap for adequate surgical planning. Intraoperative detection modalities span from 1D probes to 2D portable cameras and 3D freehand imaging.

**Conclusion:**

After successful application of radio-guided SNB and SPECT, innovation is leaning towards hybrid modalities, such as hybrid tracer and fusion of imaging approaches including SPECT/CT and PET/CT. Robotic surgery, as well as augmented reality and virtual reality techniques, is leading to application of these innovative technologies to the clinical setting, guiding surgeons towards a precise, personalised, and minimally invasive approach.

## Introduction

**Fig. 1:**
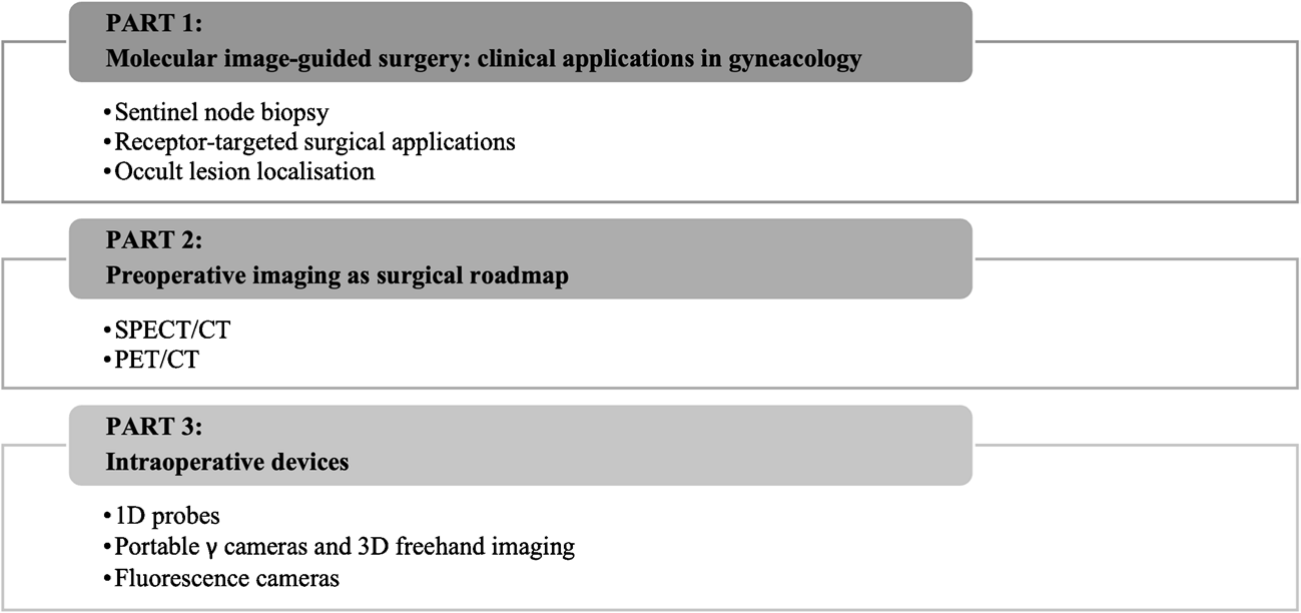
Clinical applications and modalities of image-guided surgery in gynaecological oncology

In cancer surgery, the oncological outcome depends on the radical removal of the disease. At the same time, it is crucial to limit the burden of complications and morbidity. Currently, it is possible to provide personalised and minimally invasive solutions for selected patients who will have a real benefit from surgery with reduced side effects, or can be spared from surgery when side effects outweigh the surgical benefit. In this setting, molecular image–guided surgery allows the detection of diseases through the molecular properties of employed agents or, in case of sentinel node (SN) mapping, through the physiological characteristics of the lymphatic system [1–3]. The success of image-guided surgery essentially depends on the choice of imaging agents and that of imaging technologies. The most common agents are optical or radioisotope based [1, 2]. In gynaecological cancer, the reference clinical application is sentinel node biopsy (SNB), guided by visible dyes or radioactive (both γ- and β-emitter agents), fluorescent, magnetic, and most recently hybrid signal. This procedure permits the identification of the first draining node(s) from the primary tumour, thereby providing diagnostic information on nodal status and reducing the risk of post-operative morbidity in case of non-metastatic SN(s) [4, 5]. Preoperative target detection is a valid adjunct to surgical planning. Traditional lymphoscintigraphy has been implemented by SPECT/CT allowing accurate SN mapping, being particularly useful in complex anatomical areas [3, 6]. Similarly, intraoperative detection of radiopharmaceuticals has evolved from uni- and bi-dimensional detection systems to innovative three-dimensional freehand imaging, providing intraoperative roadmaps [7]. Using indocyanine green (ICG), instead, signal can be detected by fluorescent cameras and near-infrared (NIR) fluorescent probes offering real-time optical identification [8]. Finally, the recent implementation of tracers, devices and technologies enables to target and resect lesions using a minimally invasive robotic surgery. Also in this setting, molecular imaging permits a preoperative roadmap for adequate surgical planning. Moreover, molecular imaging can be directly visualised on the surgeon’s display for intraoperative target navigation detection and excision [1, 9]. This review aims to discuss the state-of-the-art of molecular image-guided surgery in gynaecological malignancies, from both clinical and technological points of view, with a focus on the most recent advances in nuclear medicine regarding new radiopharmaceuticals and new imaging modalities in comparison to alternative approaches and a hint to future directions (Fig. 1).

## Part 1: Molecular image–guided surgery: clinical applications in gynaecology

### Sentinel node biopsy

SNB is a minimally invasive technique which allows the identification of the first node(s) draining the primary tumour. It aims to detect micrometastases, as well as macrometastases not visualised on preoperative imaging, thus avoiding extensive nodal resections and reducing risk of post-operative morbidity [4, 5]. Depending on the selected tracer, it can be accompanied by preoperative imaging in what is commonly referred as SN mapping or lymphatic mapping, which allows an accurate preoperative surgical planning, based on which surgical procedure and resection are performed [3, 6].

### Tracers and dyes

SNB can be guided by visible dyes or tracers that can emit radioactive, fluorescent, magnetic, and most recently hybrid signal.

#### Blue dyes

Visible blue dyes, as patent blue or methylene blue, were proposed in the 1990s, during the early years of SNB. These dyes are still standard of care in several institutions worldwide, even though the use of blue dyes alone has been decreasing since the beginning of the 2000s due their low detection rate when compared to radioactive tracers alone or the combined use of both (68.7% vs 97.7% in vulvar cancer; 80.9% vs 92.3% in cervical cancer) [10, 11].

#### Radio-guided

The most used radiopharmaceuticals for lymphatic mapping and SNB are ^99m^Technetium (^99m^Tc)-based agents, specifically radiolabelled colloids, owing to their physical characteristics, low cost, and large availability as well as low radiation dose to the patient and the surgical staff [4, 7]. Among them, [^99m^Tc]Tc-sulphur colloids are routinely used in the USA, while [^99m^Tc]Tc-nanocolloidal albumins are most used in Europe and [^99m^Tc]Tc-antimony trisulphide in Canada and Australia. They differ in particle size, and the choice in clinical practice is mostly based on their availability and local licensure. Their pharmacological aspects and injection modalities are well described in the current EANM guidelines [4]. [^99m^Tc]Tc-tilmanocept, a new generation radiotracer, selectively binds to mannose receptors expressed on the surface of nodal macrophages and dendritic cells [12]. This radiopharmaceutical shows a rapid clearance from the injection site and a rapid visualisation of tumour-draining lymph nodes. Additionally, it has the advantage of prolonged retention in the SNs with decreased spill to second echelon nodes. Its use is expanding in several countries worldwide [13]. Also, PET radiopharmaceuticals, which are traditionally employed for diagnostic imaging, may be useful for surgical guidance in selected cases [14, 15]. Interstitial [^18^F]FDG injection into the uterine cervix has been proposed to preoperatively visualise SNs and detect tumour-positive lymph nodes, a method called positron lymphography (see section “PET/CT” in Part 2 of this paper) [14].

#### Fluorescent-guided

In the last years, fluorescence-based guidance has been explored as a useful alternative to radio-guided SNB. These agents emit electromagnetic radiation in the energy range of the NIR light spectrum. These fluorophore agents are not visible to the human eye, but they are intraoperatively detected through a fluorescence detection system [16]. They allow signal detection using non-ionising radiation — thus not subject to the radiation safety rules — with the use of portable detection systems, such as fluorescence 1D-probes, cameras, or even 3D freehand surface imaging [17]. However, their main disadvantage is represented by the limited depth of penetration of the light in human body, in particular in fatty tissue, which could reduce its intraoperative sensitivity when lying behind other anatomic structures [18]. For the same reason, the use of fluorescence agents does not always allow preoperative imaging and planning. In the realm of fluorescence, ICG is the most used example of non-targeted fluorescent dye. It must be highlighted that the role of fluorescence in gynaecology is not limited to SNB. Indeed, it can also represent an aid for the surgeons to reduce intraoperative damage and morbidity allowing florescent guidance in dynamic processes, e.g., identifying ureters or pelvic nerves, helping to verify bowel perfusion after re-anastomosis, or vascular perfusion and the potential viability of a reconstruction flap [19–24]. This is made possible by using fluorescent tracers to mark other tissue than SNs, which can be easily intraoperatively identified, making oncologic pelvic surgery safer.

#### Hybrid signal

To overcome the limitations of ICG, the so-called “hybrid tracers” have been developed in the last decade, which combine radioactive and fluorescent guidance in a single injection [25]. The main strength point of hybrid tracers is to obtain the preoperative imaging roadmap by radiopharmaceutical guidance and real-time intraoperative visualisation of SNs by fluorescent guidance, thus realising “the best of both worlds” [2, 26]. The first hybrid tracer was ICG-[^99m^Tc]Tc-nanocolloid, which is currently the most widely available and the most used for clinical applications [27–29]. Indeed, other tracers combining radioactive and fluorescent labels have been proposed (e.g., [^99m^Tc]-labeled Cy7 tilmanocept in mice study [30]).

#### Magnetic guidance

On the side of magnetic particles, superparamagnetic iron oxides (SPIONs) were introduced for preoperative SNB mapping in the first years of 2000 and then applied for molecular image-guided surgery [31]. Magnetic tracers, such as SPIONs or lately magnetic nano-carbons, are rarely employed in gynaecology, mainly due to the limited availability of the tracer and detecting devices. Yet, the first studies reported their use in cervical, vulvar, and endometrial cancers evaluating preoperative SN mapping using magnetic tracers [32–34].

### Clinical scenarios

Nodal involvement is an important prognostic factor in patients with vulvar, cervical, endometrial, and ovarian cancer, being related to reduced survival and increased recurrence rate [35–38].

#### Vulvar cancer

In vulvar cancer, the major pathway of spread is to the inguinofemoral lymph nodes and then to the pelvic ones [39, 40]. According to current guidelines, SNB is recommended for unifocal vulvar tumours (< 4 cm) with clinically negative lymph nodes (cN0), as well as for vulvar melanoma. The combination of radiotracer and visual dye (blue dye or ICG) is preferred due to the increased detection rate [41–43]. The pioneering clinical trials were represented by the GOG-173 and GROINSS-V study, which validated this procedure as an alternative to extended lymphadenectomy in selected cases using radiotracer and blue dye [44, 45]. The GROINSS-V study also demonstrated that the tumoural load in the SN was strongly associated with survival [46]. Most recently, long-term results of the GROINSS-V reported a 5-year isolated groin recurrence rate of 2.5% for patients with negative SNB and of 8.0% for positive SNB [47]. Moreover, the GroSNaPET study by Garganese et al. reported SNB safety (none false negative) in cases currently excluded from the procedure, such as: patients with *T* > 4 cm or multifocal tumours; after complete primary lesion diagnostic excision; in case of contralateral nodal involvement or of local recurrences*.* These results also showed that a careful preoperative work-up by [^18^F]FDG PET/CT is critical in the selection of cases with cN0 [48, 49]. Concerning fluorescent-guided SNB, the first clinical results showed feasibility with ICG [8]. Subsequent studies, including a recent systematic review, confirmed safety of this technique, with a SN detection rate ranging from 89.7 to 100% [50]. Clinical studies using the hybrid tracer ICG-[^99m^Tc]Tc-nanocolloid underlined how the addition of fluorescence guidance could improve visualisation with respect to blue dyes in vulvar cancer [29, 51]. A multicentre randomised controlled trial could confirm these results as a significantly higher number of resected SNs were fluorescent- (92.5%) than blue-stained (65.3%) [52].

#### Cervical cancer

In cervical cancer, lymphatic drainage principally occurs to pelvic nodes (parametrial, internal/external iliac, and presacral) and subsequently to common iliac and para-aortic regions [53]. According to current guidelines, SNB may be considered in the early stages. Indeed, in these patients, pelvic lymphadenectomy may be considered as an overtreatment, due to low incidence of positive nodes and high risk of surgical complications. ICG is the preferred tracer providing similar intraoperative bilateral detection rate than the combination of radiotracer and blue dye [54, 55]. The pioneering studies with radiotracer and blue dye showed high rates of SN detection (97.8%), high sensitivity (92%), and high negative predictive value (NPV, 98.2%) for detection of metastases, with bilateral detection rate of 76.5%. The usefulness of SNB for uncommon drainage patterns was also highlighted [56–58]. The SENTICOL-2 study, involving 206 early cervical cancer patients, compared SNB with radiotracer and blue dye with respect to pelvic lymph node dissection. The SNB group displayed significantly lower lymphatic morbidity (31.4% vs 51.5%) and lower post-operative neurological symptoms (7.8% vs 20.6%). Moreover, no significant differences in the 3-year recurrence-free survival were reported between the two groups [59]. Concerning fluorescent-guided SNB, the FILM trial involving 163 patients with stage I cervical or endometrial cancer, demonstrated a significant higher overall detection rate for ICG with respect to blue dye (98% for ICG vs 76% for blue dye) [60]. In the pilot study by Parades et al. using ICG-[^99m^Tc]Tc-nanocolloid in 16 early-stage cervical cancer, the hybrid tracer allowed bilateral detection in all patients with higher detection rate than blue dye [27]. In the pilot study by Murakami et al., SN were labelled using SPIONs. In five of 15 patients, radioisotope labelling was also used, with similar results between the two modalities [32].

#### Endometrial cancer

For endometrial cancer, nodal dissemination occurs to internal, external, common iliac, presacral, and para-aortic lymph nodes, with metastases being uncommon in the para-aortic region [61]. According to NCCN guidelines, SNB may be considered in apparent uterine-confined tumours without metastases at imaging nor extrauterine disease at exploration, including high risk histologies (serous carcinoma, clear cell carcinoma, carcinosarcoma). According to ESGO/ESTRO/ESP joint guidelines, SNB can be considered in patients with low- and intermediate‐risk disease and can be omitted in patients without myometrial invasion. As for cervical cancer, ICG is the preferred tracer providing similar intraoperative bilateral detection rate than the combination of radiotracer and blue dye [38, 62]. In the SENTI-ENDO prospective trial, SNB was performed through radiotracer and blue dye in 125 patients, suggesting the utility of SNB in patients with low- and intermediate-risk endometrial malignancies, with sensitivity of 84% and a NPV of 97% per patient analysis [63]. The FILM trial confirmed that ICG was able to identify more SNs than blue dye also for uterine cancers [60]. The SENTOR study, involving 156 patients with intermediate and high-grade endometrial cancer, showed that even in high-risk cases, SN by ICG mapping instead of radical lymphadenectomy appears to be a valid option. They reported a bilateral detection rate of 77.6%, sensitivity of 96%, and a NPV of 99% [64]. In 52 patients with intermediate and high-risk endometrial cancer, the use of the hybrid tracer ICG-[^99m^Tc]Tc-nanocolloid showed sensitivity and NPV for metastases of 100%. Moreover, the increased detection rate of para-aortic SNs showed the potential to become a promising alternative also for uterine tumours [28].

#### Ovarian cancer

Ovarian cancer can disseminate along three main pathways: peritoneal, lymphatic, and hematogenous [65]. Concerning nodal involvement, para-aortic and pelvic lymph nodes are the most common sites of spread [66, 67]. In malignant ovarian tumour, SNB is not routinely performed and recognised in clinical practice, but some authors have assessed its feasibility. Injections are mainly performed and recommended in the ovarian ligaments, during laparoscopic or open surgery [68, 69]. Feasibility studies with blue dye and [^99m^Tc]Tc-nanocolloid assessed safety of the procedure, with SNs visualised in all cases, yet highlighting the need of further evidence to confirm the results (96% rate of successful procedure; detection rate of 100%) [70, 71]. In the SENTOV phase II clinical trial including 20 patients with early-stage ovarian cancer, SNB by ICG and [^99m^Tc]Tc-nanocolloid showed a detection rate of 93% for pelvic SNs and 100% for para-aortic SNs. No adverse events were intraoperatively reported, nor within 30 days, suggesting safety of the approach [72, 73]. The SELLY phase II prospective interventional trial is currently ongoing, evaluating performance, feasibility, and safety of SNB performed with ICG in early-stage ovarian cancer patients [74].

### Receptor-targeted surgical applications

Among molecular imaging approaches, systemic administered targeted tracers represent a promising modality. They are constituted by a radioactive or fluorescent probe and by a carrier, that can be a small molecule, an antibody or a peptide [18]. In this way, they represent a highly specific molecular marker of disease that can be exploited as targeted cancer biomarker [75]. Their application in gynaecology is mainly based on the expression of folate receptor-α (FR-α) which is overexpressed in ovarian cancer, especially in high-grade serous histotypes [76], and in endometrial adenocarcinoma [77]. Among the first human studies, FR-α has been explored as a target for ovarian lesions through different fluorescent tracers: folate-FITC [78], EC17 [79], and OTL38 [80]. Among the first human studies, folate-FITC was employed in a pilot study on ten patients with ovarian tumours. This tracer was demonstrated to detect malignant lesions with FR-α expression, displaying also small metastases (< 1 mm). Conversely, malignant lesions without FR-α expression or benign tumours were not detectable [78], EC17 was assessed for intraoperative fluorescence imaging in 12 ovarian cancer patients. It resulted in a clear signal in malignant tissues, accomplishing resection of additional lesions not detected with inspection or palpation. However, several limitations were pointed out, as the low tissue penetration and reduced detection below the surface. Moreover, 23% of lesions were identified as false positives. This drawback could be explained by the collagen-containing structures, that can emit autofluorescent signals [79]. Similarly, OTL38, another FR-α binding agent, was assesses in 12 patients with ovarian cancer. It allowed a clear fluorescent signal and detection of the lesions. A percentage of 29% of additional malignant lesions were identified, that were otherwise undetectable [80].

OTL38 also allowed the detection of endometrial cancer lesions together with nodal and omental metastases. Nonetheless, false positives lymph nodes were reported, caused by FR-β expression by activated macrophages [81]. More recently, phase-II and phase-III trials have assessed OTL38 in larger ovarian cancer cohorts, showing that this tracer could offer real-time detection of further lesions. These results show the potential of intraoperative fluorescence in these tumours, improving resection accuracy and reducing the burden of residual disease [82, 83]. However, no study so far has shown a survival benefit when utilising these tracers. Folate conjugates have been assessed also in combination with SPECT and PET tracers for nuclear medicine imaging [84]. Among them, human studies have investigated the use of ^111^In-DTPA-folate in ovarian cancer patients [85], and ^99m^Tc-EC20 in multiple solid tumours, such as ovarian and endometrial cancer [86]. Concerning radiolabelled folates for PET imaging, to our knowledge, no human study results have yet been reported, but clinical trials are ongoing (NCT05215496 and NCT03242993). Indeed, due to the promising potential of folate-based PET radiopharmaceuticals, we expect the development of further studies in the foreseeable future. Finally, following a similar rationale as in the case of SNB, hybrid receptor-targets are under development, such as ^111^In-farletuzumab-IRDye800CW that targeted FRα successfully in mice with ovarian cancer [87].

### Occult lesion localisation

**Fig. 2:**
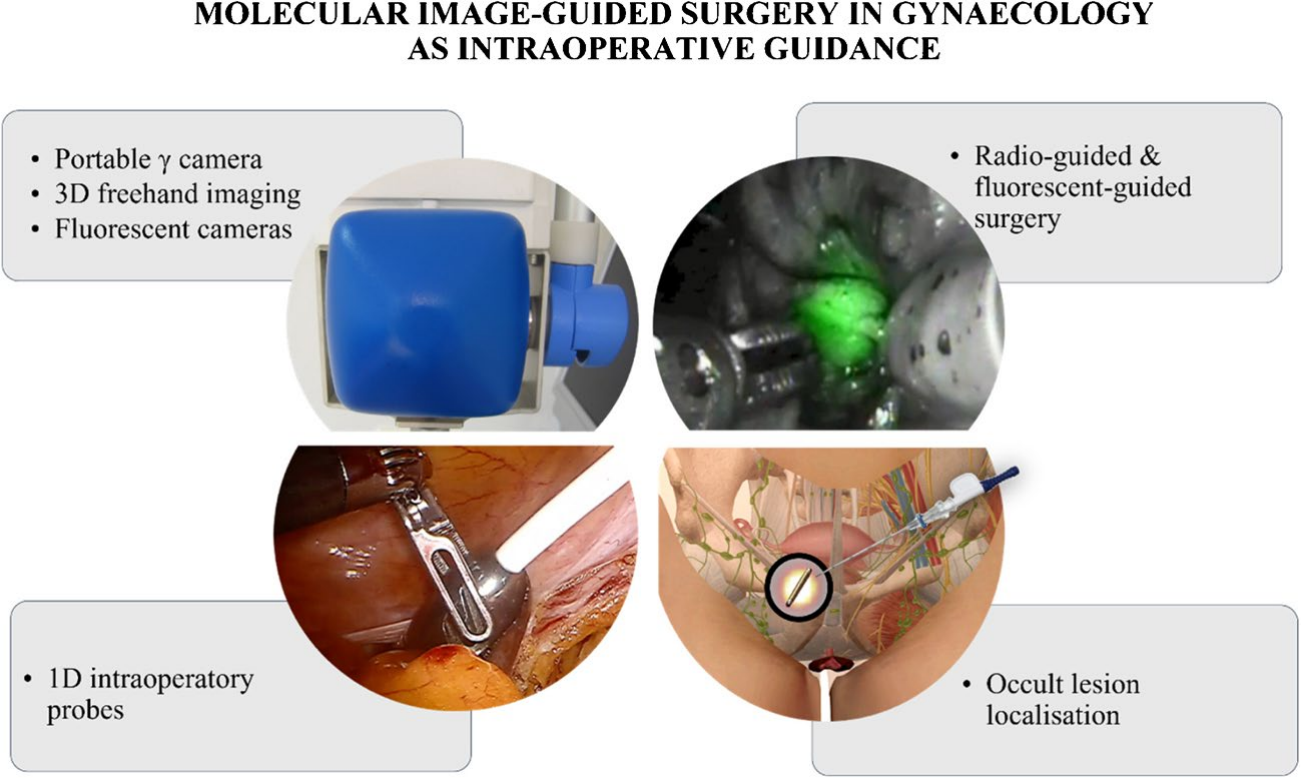
Some examples of the possible applications of molecular image–guided surgery in gynaecology to aid and improve surgical guidance

Occult lesion localisation techniques use radiopharmaceuticals (e.g., [^99m^Tc]Tc-MAA or ^125^I) to mark tumours otherwise not detectable in the operating room (Fig. 2). The most common are radio-guided occult lesion localisation (ROLL) and radio-guided seed localisation (RSL), which rely on the injection of a radiopharmaceutical in the target lesion to enable radio-guided excision. Yet, the use of radioactivity as a tracer can be replaced by magnetic seeds or/and radio-frequency identification chip as used in breast cancer [88, 89]. Evaluations in gynaecologic malignancies have been very limited. The ROLL procedure may hold potential for detection of peritoneal lesions. In a patient with uterine leiomyosarcoma, [^99m^Tc]Tc-MAA injection allowed radio-guided excision of the peritoneal metastases, facilitated also by the anatomical SPECT/CT localisation [90]. Among the potential applications, RSL through ^125^I-seeds has been performed with success for non-palpable soft tissue masses [91]. Furthermore, a pilot study assessed feasibility of RSL for preoperative localisation of suspicious non-palpable lymph nodes, including one case with vulvar melanoma. They achieved a successful excision in all cases, suggesting the feasibility of this approach also for gynaecological cancers [92].

## Part 2: Preoperative imaging as surgical roadmap

Radioactive, magnetic, and hybrid tracers show a major advantage over pure fluorescent ones, as they enhance preoperative imaging. Most literature focuses on SPECT/CT and PET/CT; nevertheless, these results may possibly be extended to MRI when using magnetic tracers.

### SPECT/CT

Preoperative SPECT/CT allows the detection of more SNs than 2D planar lymphoscintigraphy. Moreover, it gives an anatomical correlation and reduces the false positive findings such as external contamination or by uptake in enlarged lymphatic vessels [39, 93–96]. In detail, it enables for better contrast and spatial resolution, showing the morphologic features of the target in 3D relationship to anatomical structures and landmarks. This aspect may be advantageous in cases of complex lymphatic drainage and anatomy [97]*.* In vulvar cancer, preoperative SPECT/CT can visualise more SNs with respect to planar lymphoscintigraphy and with better anatomical localisation. It is particularly useful in localising lymph nodes in the corresponding Daseler zone (83% in the medial region), or in case of nodal migration to unexpected sites (i.e., 17.5% in vesical, paravesical, retrovesical, paravaginal, and gluteal) [39, 95]. Also for cervical and endometrial malignancies, SPECT/CT enables a better bilateral detection with respect to planar lymphoscintigraphy (e.g., 69.0% vs 66.7% in cervical cancer, respectively) [96, 97]. In addition, this modality facilitates identification of SNs close to the injection point or in uncommon sites, such as para-aortic and pre-sacral regions [98–100]. Hence, there are preliminary experiences on fusion virtual navigation, which use molecular imaging to guide ultrasound for invasive diagnostic procedures on target inguinal nodes previously highlighted by SPECT/CT in vulvar cancer patients [101].

### PET/CT

**Fig. 3:**
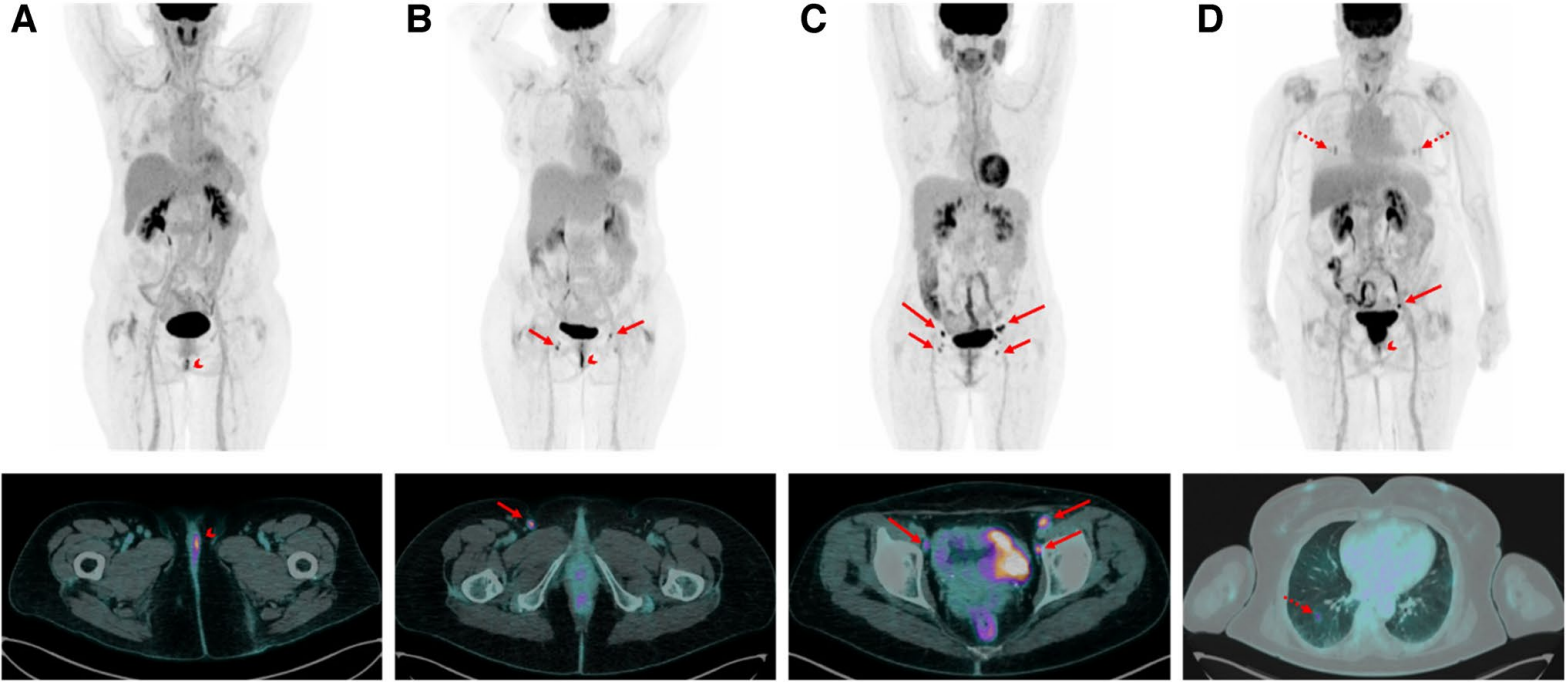
Multiple intensity projection (MIP) (top) and transverse fused PET/CT images (bottom) of four vulvar cancer patients. **A** A 66-year-old woman with left-side unilateral vulvar squamous cell carcinoma (SCC) of 2.5 cm diameter. PET/CT images showing [^18^F]-FDG uptake just in the primary tumour (arrowhead). This patient was scheduled for partial vulvectomy and ipsilateral SNB. **B** A 51-year-old woman with midline vulvar SCC of 3 cm diameter. PET/CT images showing [^18^F]-FDG uptake corresponding to the primary tumour (arrowhead) and focal [^18^F]-FDG uptake in bilateral inguinal lymph nodes (short arrows). This patient was scheduled for radical vulvectomy and bilateral inguinal lymphadenectomy. **C** A 65-year-old woman with midline vulvar SCC of 3 cm diameter previously excised. PET/CT images showing focal [^18^F]-FDG uptake in bilateral inguinal lymph nodes (short arrows) and bilateral pelvic nodes, which were located in the obturator and external iliac regions (long arrows). This patient was scheduled for upfront chemo-radiotherapy. **D** An 82-year-old woman with vulvar melanoma. MIP showing focal [^18^F]-FDG uptake in the primary vulvar tumour (arrowhead), in the left internal iliac node (long arrow) and bilateral pulmonary nodules (dashed arrows). Transverse fused PET/CT images showing focal [^18^F]-FDG uptake corresponding to a sub-centimetric pulmonary nodule localised in the right inferior lobe. This patient was scheduled for systemic therapy

Current guidelines consider [^18^F]FDG PET/CT for initial work-up in patients affected by uterine, cervical, and vulvar cancer with locally advanced tumour or in selected cases when metastatic disease is suspected [38, 41, 54]. Limited data are available on the role of [^18^F]FDG PET/CT in nodal staging of patients with early stage disease [102–104]. A potential clinical impact may be anticipated for vulvar cancer [105]. A recent systematic review and meta-analysis, collecting the results of few studies on small series of vulvar cancer patients, showed a good NPV (92%) and a disappointing PPV (70%) with an overall prevalence of metastatic groin of 28.6% [106]. Indeed, a positive lymph node is not highly predictive of metastasis as inguinal reactive lymph nodes can concentrate [^18^F]FDG. Specificity is still suboptimal even when delayed imaging at 3 h from [^18^F]FDG injection is used [49]. The high NPV of preoperative [^18^F]FDG PET/CT was confirmed in a large retrospective study of 160 vulvar cancer patients [107]. In the GroSNaPET study, preoperative [^18^F]FDG PET/CT was investigated in cN0 patients currently unfit for SNB. This study highlighted a high NPV (93%). Importantly, all false negative cases at [^18^F]FDG PET/CT were identified by SNB and no false negative SNs were found. These results suggest that the combined use of [^18^F]FDG PET/CT and SNB is useful to better select cN0 patients who could benefit for a less invasive surgical treatment or for sparing adjuvant radiation therapy, thus reducing treatment toxicity [48]. Additionally, a positive [^18^F]FDG PET/CT may guide therapeutic decision making (Fig. 3). New methods to employ PET tracers for preoperative mapping have been attempted. In a pilot clinical trial, patients with cervical or uterine cancer underwent interstitial [^18^F]FDG injections in the uterine cervix (positron lymphography). Dynamic PET/CT and late-phase PET/CT were carried out to detect metastatic invasion in [^18^F]FDG-avid lymph nodes. Afterwards, patients underwent standard staging surgery and SNB with blue dye or ICG. The procedure demonstrated a high sensitivity, even though further evidence is warranted [14]. Another approach to integrate PET/CT within a surgical navigation setup is the use of PET/CT combined with an intraoperative ultrasound. In the study by Garganese et al. including breast cancer and gynaecological cancer patients, the pre-acquired [^18^F]FDG PET/CT images were uploaded as Digital Imaging and Communications in Medicine (DICOM) files and registered with real-time ultrasound, performed on the superficial nodes. The results showed that fusion could be successfully achieved, detecting the suspicious target lymph nodes in most cases. Moreover, this technique could be used to guide bioptical procedures, as well as for diagnostic or therapeutic approaches [15]. In addition to [^18^F]FDG, Fibroblast Activation Protein Inhibitor (FAPI) labelled with Gallium-68 is emerging as a promising radiotracer for PET/CT in gynaecological cancers [108, 109]. Its potential role for preoperative roadmap in these malignancies deserves to be investigated.

## Part 3: Intraoperative devices

Intraoperative detection modalities span from 1D probes to 2D portable fluorescent or γ cameras and 3D freehand imaging, such as freehand SPECT (Fig. 2). Localising tracer-labelled structures in the operating room requires sterile fashion (open setting) or rather small detectors to be placed inside the surgical incision (laparoscopic or robotic surgery). Herein, we present the most common devices used in molecular image-guided surgery.

### 1D probes

The radioactive targets can be detected in real-time during surgical operations through handheld nuclear probes. They consist of a 1D detection approach that measures the emitted γ or β particles. Signal intensity is measured in counts per second with a small field of view, providing audible and numerical signals in open surgery [7]. Similarly, 1D fluorescence and magnetic probes have been proposed to detect fluorescent or magnetic tracers.

### Nuclear probes

Gamma-emitting radiotracers emit γ photons that can be detected through low-to-mid energy probes. Their most common applications are SNB with [^99m^Tc]Tc-nanocolloid [110–113]. PET tracers can be detected through high-energy γ-probes, for precise surgical excision of lesions characterised by high [^18^F]FDG uptake, such as cervical, endometrial, and ovarian carcinoma [114]. PET isotopes can also be detected using β + probes. A prospective study exploited these probes for ex vivo evaluation of cervical cancer specimens [115]. Additionally, in cervical cancer patients, a surgical scenario by a Monte Carlo simulation of the β probe suggests that this approach could help surgeons to distinguish tumour margins from healthy tissue [116]. From the handheld probes for open surgery [111, 112, 117], technology is progressively moving in the direction of less invasive approaches as (robotic-assisted) laparoscopic surgery. Rigid and elongated γ laparoscopic probes were developed to be inserted in the trocar. Nonetheless, the limited rotational freedom makes it difficult to reach the target in proximity to high signal backgrounds. To increase the manoeuvrability, a tethered DROP-IN γ-probe was developed for robotic-assisted laparoscopic surgery [9]. After being first tested for prostate cancer [118], it has been used also in the setting of uterine cervix malignancies during robotic surgery. In a feasibility study by Baeten et al., ten patients with cervical cancer were included. They underwent SNB with [^99m^Tc]Tc-nanocolloids, preoperative imaging and robot-assisted laparoscopic intervention. For SN localisation, a tethered DROP-IN γ-probe was employed; then, a rigid laparoscopic probe was used to confirm the results. No significant difference resulted from the count rate of the two probes, as well as no adverse events were recorded [119]. The combination of DROP-IN γ probes and freehand SPECT has been also shown in a phantom setup, called in-patient SPECT [120]. These developments may gain further momentum given the recent advantages in machine learning-based techniques for tracking [121]. To gain yet more flexibility, the DROP-IN γ probe has been modified to be mounted directly on a grasper either in a laparoscopic or a robotic surgery setup. This concept is called the CLICK-ON γ probe and is promising towards reducing the learning curve of surgeons [122].

### Fluorescence and magnetic probes

Unlike radioactive tracers, fluorescent and magnetic tracer detection needs specific stimulation. For fluorescence, next to the detector, a light fibre is placed to excite the fluorescence commonly with a laser light [7]. When using hybrid tracers, an opto-nuclear probe allows both the detection of NIR fluorescent and γ-photons. This approach, which has been tested in patients with various tumours including cervical cancer, was shown to be feasible both for open and laparoscopic surgery [123]. For magnetic probes, an electromagnetic field is generated by the probe and its distortion is used to detect the amount of magnetic tracer in the vicinity [124, 125]. To our knowledge, these types of probes have not yet been used in gynaecology. However, in the frame of hybrid tracers, combined γ and fluorescence probes are also available and have been evaluated in endometrial cancer [126].

### Portable γ cameras and 3D freehand imaging

When radiopharmaceuticals are employed, 2D images can be acquired using portable γ cameras, that display radioactivity uptake distribution during surgical navigation [1]. This modality can be useful in the case of deep lesions, overlapping of targets such as the injection site and SNs, or to check the surgical field after excision of the target lesion [7]. The use of this modality has been exploited in the SN excision in cases of pelvic tumours. In particular, the possibility to compare real-time intraoperative findings with the preoperative SPECT/CT is useful in laparoscopic surgery, especially for iliac and para-aortic nodes [3, 127]. Nevertheless, the size of the detector, the limited manoeuvrability, and the costs have shifted use in favour of probes or conventional preoperative imaging [3, 128, 129].

To obtain 3D imaging, the concept of freehand SPECT has been developed [1]. The freehand SPECT technique may be particularly useful for internal organs, in which tracer administration is less accessible or 3D information is highly valuable, like uterine malignancies. In addition, the complex lymphatic drainage of these tumours can be challenging during laparoscopic procedures. In patients with cervical and endometrial cancers, freehand SPECT was employed to identify SNs and guide their excision, providing a real time 3D image, which was useful to guide surgeons to the target [130].

Fusion of freehand SPECT with ultrasound has been attempted in patients with various malignancies, including vulvar cancer. Co-registration of the hot spot with the lymph node visualised by ultrasound was achieved with a good rate of success, showing the potential usefulness of the procedure [131]. The concept of freehand SPECT can be also extended to other modalities. For instance, instead of using a 1D γ probe, 3D surface images can be generated from 1D fluorescence probes as well as 1D magnetic probes [132]. The technology of freehand SPECT was initially developed for 3D surface imaging of beta radiation [133].

### Fluorescence cameras

Fluorescence cameras provide real-time 2D images of in vivo fluorescent tracer distributions. Varying from open-surgery devices to laparoscopic ones, they use optical filters to display only the fluorescent light emitted by the tracer, cancelling any ambient light and most importantly the fluorescence excitation light. Newer and more complex setups include the possibility to acquire a white-light image next to the fluorescence one, to better understand the anatomical localisation of the fluorescent signal. A custom-made multispectral fluorescence camera system for intraoperative use was reported in vulvar cancer patients, undergoing SNB with ICG and blue dye [8].

## Future directions

Robotic surgery is gaining momentum in the field of minimally invasive surgery in gynaecological malignancies. Robot assisted SNB has been performed in vulvar, cervical and endometrial cancer patients, showing feasibility and potential to decrease short and long-term morbidity [134–136]. In addition, recent advances have let the integration of molecular imaging within the laparoscopic view of the surgeon. For instance, the digital environment created by virtual reality can be exploited pre- or intraoperatively, highlighting the anatomical structures and targets in relation with the surgical tools. Augmented reality visualisations allow the display of images directly on the patient, in the real surgical context [1]. The drawbacks of these technologies, such as poor registration accuracy and risk of improper interpretation, have reduced enthusiasm for such an approach. Nonetheless, technological improvements through artificial intelligence could overcome these limits and support their clinical application [137]. These approaches have been mostly used in gynaecological settings for benign pathologies, such as for dissection of uterine myomas. They have shown potential to guide the surgeon in cases of small or medium-sized myomas, that are difficult to detect [138]. Most recently, a phantom model was built to attempt real-time co-registration of preoperative SPECT with intraoperative CT, in order to integrate augmented reality guidance during SNB in endometrial cancer patients [139]. This preliminary work may pave the way for application of these technologies in gynaecological clinical setting. Finally, financial aspects require a careful evaluation. To our knowledge, no studies described cost-effectiveness and cost-utility of image-guided surgery technologies in gynaecological cancers, comparing for instance different tracers and devices. These would be essential to allow reimbursement, and consequently enable their widespread use.

## Conclusions

In the field of gynaecology, molecular image–guided surgery is increasingly used for a precise surgical guidance to increase resection accuracy and minimise surgical risk, thus improving patient outcome and quality of life. After successful application of radio-guided SNB and SPECT, innovation is leaning towards hybrid modalities, such as the hybrid tracer ICG-[^99m^Tc]Tc-nanocolloid and the fusion of imaging approaches including SPECT/CT and PET/CT. Robotic surgery, as well as augmented reality and virtual reality techniques, is leading to application of these innovative technologies to the clinical setting, guiding surgeons towards a precise, personalised, and minimally invasive approach. Financial aspects might represent a limit in the clinical work-up; thus, further studies are needed to investigate the cost-effectiveness of both innovative agents and devices.

## Data Availability

All data used in the current manuscript can be retrieved from the respective references.

## References

[1] Wendler T, van Leeuwen FWB, Navab N, van Oosterom MN. How molecular imaging will enable robotic precision surgery: the role of artificial intelligence, augmented reality, and navigation. Eur J Nucl Med Mol Imaging. 2021;48:4201–24. 34185136 10.1007/s00259-021-05445-6 PMC8566413

[2] van Leeuwen FWB, Schottelius M, Brouwer OR, Vidal-Sicart S, Achilefu S, Klode J, et al. Trending: radioactive and fluorescent bimodal/hybrid tracers as multiplexing solutions for surgical guidance. J Nucl Med. 2020;61:13–9. 31712326 10.2967/jnumed.119.228684

[3] Valdés Olmos RA, Rietbergen DDD, Rubello D, Pereira Arias-Bouda LM, Collarino A, Colletti PM, et al. Sentinel node imaging and radioguided surgery in the era of SPECT/CT and PET/CT: toward new interventional nuclear medicine strategies. Clin Nucl Med. 2020;45:771–7. 32701805 10.1097/RLU.0000000000003206

[4] Giammarile F, Bozkurt MF, Cibula D, Pahisa J, Oyen WJ, Paredes P, et al. The EANM clinical and technical guidelines for lymphoscintigraphy and sentinel node localization in gynaecological cancers. Eur J Nucl Med Mol Imaging. 2014;41:1463–77. 24609929 10.1007/s00259-014-2732-8

[5] Oonk MHM, van de Nieuwenhof HP, de Hullu JA, van der Zee AGJ. The role of sentinel node biopsy in gynecological cancer: a review. Curr Opin Oncol. 2009;21:425–32. 19593136 10.1097/CCO.0b013e32832f3d53

[6] Collarino A, Fuoco V, Garganese G, Pereira Arias-Bouda LM, Perotti G, Manca G, et al. Lymphoscintigraphy and sentinel lymph node biopsy in vulvar carcinoma: update from a European expert panel. Eur J Nucl Med Mol Imaging. 2020;47:1261–74. 31897584 10.1007/s00259-019-04650-8

[7] Van Oosterom MN, Rietbergen DDD, Welling MM, Van Der Poel HG, Maurer T, Van Leeuwen FWB. Recent advances in nuclear and hybrid detection modalities for image-guided surgery. Expert Rev Med Devices. 2019;16:711–34. 31287715 10.1080/17434440.2019.1642104

[8] Crane LMA, Themelis G, Arts HJG, Buddingh KT, Brouwers AH, Ntziachristos V, et al. Intraoperative near-infrared fluorescence imaging for sentinel lymph node detection in vulvar cancer: first clinical results. Gynecol Oncol. 2011;120:291–5. 21056907 10.1016/j.ygyno.2010.10.009

[9] Meershoek P, van Oosterom MN, Simon H, Mengus L, Maurer T, van Leeuwen PJ, et al. Robot-assisted laparoscopic surgery using DROP-IN radioguidance: first-in-human translation. Eur J Nucl Med Mol Imaging. 2019;46:49–53. 30054696 10.1007/s00259-018-4095-z PMC6267681

[10] Meads C, Sutton AJ, Rosenthal AN, Małysiak S, Kowalska M, Zapalska A, et al. Sentinel lymph node biopsy in vulval cancer: systematic review and meta-analysis. Br J Cancer. 2014;110:2837–46. 24867697 10.1038/bjc.2014.205 PMC4056048

[11] Kadkhodayan S, Hasanzadeh M, Treglia G, Azad A, Yousefi Z, Zarifmahmoudi L, et al. Sentinel node biopsy for lymph nodal staging of uterine cervix cancer: a systematic review and meta-analysis of the pertinent literature. Eur J Surg Oncol. 2015;41:1–20. 25454828 10.1016/j.ejso.2014.09.010

[12] Vera DR, Wallace AM, Hoh CK, Mattrey RF. A synthetic macromolecule for sentinel node detection: (99m)Tc-DTPA-mannosyl-dextran. J Nucl Med. 2001;42:951–9. 11390562

[13] Rovera G, de Koster EJ, Rufini V, Zollino M, Zagaria L, Giammarile F, et al. 99mTc-Tilmanocept performance for sentinel node mapping in breast cancer, melanoma, and head and neck cancer: a systematic review and meta-analysis from a European expert panel. Eur J Nucl Med Mol Imaging. 2023;50:3375–89. 37310426 10.1007/s00259-023-06290-5

[14] Mueller JJ, Dauer LT, Murali R, Iasonos A, Pandit-Taskar N, Abu-Rustum NR, et al. Positron lymphography via intracervical 18F-FDG injection for presurgical lymphatic mapping in cervical and endometrial malignancies. J Nucl Med. 2020;61:1123–30. 31924717 10.2967/jnumed.119.230714 PMC7413230

[15] Garganese G, Bove S, Fragomeni S, Moro F, Triumbari EKA, Collarino A, et al. Real-time ultrasound virtual navigation in 3D PET/CT volumes for superficial lymph-node evaluation: innovative fusion examination. Ultrasound Obstet Gynecol. 2021;58:766–72. 33587289 10.1002/uog.23613

[16] Manny TB, Patel M, Hemal AK. Fluorescence-enhanced robotic radical prostatectomy using real-time lymphangiography and tissue marking with percutaneous injection of unconjugated indocyanine green: the initial clinical experience in 50 patients. Eur Urol. 2014;65:1162–8. 24289911 10.1016/j.eururo.2013.11.017

[17] van Oosterom MN, Meershoek P, Welling MM, Pinto F, Matthies P, Simon H, et al. Extending the hybrid surgical guidance concept with freehand fluorescence tomography. IEEE Trans Med Imaging. 2020;39:226–35. 31247546 10.1109/TMI.2019.2924254

[18] Schouw HM, Huisman LA, Janssen YF, Slart RHJA, Borra RJH, Willemsen ATM, et al. Targeted optical fluorescence imaging: a meta-narrative review and future perspectives. Eur J Nucl Med Mol Imaging. 2021;48:4272–92. 34633509 10.1007/s00259-021-05504-y PMC8566445

[19] Raimondo D, Borghese G, Mabrouk M, Arena A, Ambrosio M, Del Forno S, et al. Use of indocyanine green for intraoperative perfusion assessment in women with ureteral endometriosis: a preliminary study. J Minim Invasive Gynecol. 2021;28:42–9. 32283326 10.1016/j.jmig.2020.04.004

[20] He K, Li P, Zhang Z, Liu J, Liu P, Gong S, et al. Intraoperative near-infrared fluorescence imaging can identify pelvic nerves in patients with cervical cancer in real time during radical hysterectomy. Eur J Nucl Med Mol Imaging. 2022;49:2929–37. 35230489 10.1007/s00259-022-05686-z PMC9206623

[21] Doyle PJ, Lipetskaia L, Duecy E, Buchsbaum G, Wood RW. Sodium fluorescein use during intraoperative cystoscopy. Obstet Gynecol. 2015;125:548–50. 25730214 10.1097/AOG.0000000000000675

[22] Cabanes M, Boria F, Hernández Gutiérrez A, Zapardiel I. Intra-operative identification of ureters using indocyanine green for gynecological oncology procedures. Int J Gynecol Cancer. 2020;30:278. 31722965 10.1136/ijgc-2019-000895

[23] Moukarzel LA, Byrne ME, Leiva S, Wu M, Zhou QC, Iasonos A, et al. The impact of near-infrared angiography and proctoscopy after rectosigmoid resection and anastomosis performed during surgeries for gynecologic malignancies. Gynecol Oncol. 2020;158:397–401. 32460995 10.1016/j.ygyno.2020.05.022 PMC7693678

[24] Bizzarri N, Foschi N, Loverro M, Tortorella L, Santullo F, Rosati A, et al. Indocyanine green to assess vascularity of ileal conduit anastomosis during pelvic exenteration for recurrent/persistent gynecological cancer: a pilot study. Front Oncol. 2021;11: 727725. 34950574 10.3389/fonc.2021.727725 PMC8691262

[25] Buckle T, van Leeuwen AC, Chin PTK, Janssen H, Muller SH, Jonkers J, et al. A self-assembled multimodal complex for combined pre- and intraoperative imaging of the sentinel lymph node. Nanotechnology. 2010;21: 355101. 20689167 10.1088/0957-4484/21/35/355101

[26] KleinJan GH, van Werkhoven E, van den Berg NS, Karakullukcu MB, Zijlmans HJM a. A, van der Hage JA, et al. The best of both worlds: a hybrid approach for optimal pre- and intraoperative identification of sentinel lymph nodes. Eur J Nucl Med Mol Imaging. 2018;45:1915–25. 10.1007/s00259-018-4028-x PMC6132545 29696442

[27] Paredes P, Vidal-Sicart S, Campos F, Tapias A, Sánchez N, Martínez S, et al. Role of ICG-99mTc-nanocolloid for sentinel lymph node detection in cervical cancer: a pilot study. Eur J Nucl Med Mol Imaging. 2017;44:1853–61. 28492965 10.1007/s00259-017-3706-4

[28] Sánchez-Izquierdo N, Vidal-Sicart S, Campos F, Torné A, Angeles MA, Migliorelli F, et al. Detection of the sentinel lymph node with hybrid tracer (ICG-[99mTc]Tc-albumin nanocolloid) in intermediate- and high-risk endometrial cancer: a feasibility study. EJNMMI Res. 2021;11:123. 34905122 10.1186/s13550-021-00863-x PMC8671586

[29] Mathéron HM, van den Berg NS, Brouwer OR, Kleinjan GH, van Driel WJ, Trum JW, et al. Multimodal surgical guidance towards the sentinel node in vulvar cancer. Gynecol Oncol. 2013;131:720–5. 24051219 10.1016/j.ygyno.2013.09.007

[30] Emerson DK, Limmer KK, Hall DJ, Han S-H, Eckelman WC, Kane CJ, et al. A receptor-targeted fluorescent radiopharmaceutical for multireporter sentinel lymph node imaging. Radiology. 2012;265:186–93. 22753678 10.1148/radiol.12120638 PMC3447170

[31] Minamiya Y, Ito M, Katayose Y, Saito H, Imai K, Sato Y, et al. Intraoperative sentinel lymph node mapping using a new sterilizable magnetometer in patients with nonsmall cell lung cancer. Ann Thorac Surg. 2006;81:327–30. 16368392 10.1016/j.athoracsur.2005.06.005

[32] Murakami K, Kotani Y, Suzuki A, Takaya H, Nakai H, Matsuki M, et al. Superparamagnetic iron oxide as a tracer for sentinel lymph node detection in uterine cancer: a pilot study. Sci Rep. 2020;10:7945. 32409660 10.1038/s41598-020-64926-0 PMC7224276

[33] Jedryka MA, Klimczak P, Kryszpin M, Matkowski R. Superparamagnetic iron oxide: a novel tracer for sentinel lymph node detection in vulvar cancer. Int J Gynecol Cancer. 2020;30:1280–4. 32675253 10.1136/ijgc-2020-001458

[34] Wang YN, Xia YF, Chen YQ, Qi HY, Lou YH. Application of the two-step sentinel lymph node biopsy with double-tracer in early staged endometrial cancer. Zhonghua Fu Chan Ke Za Zhi. 2022;57:812–20. 36456477 10.3760/cma.j.cn112141-20220323-00185

[35] Burger MP, Hollema H, Emanuels AG, Krans M, Pras E, Bouma J. The importance of the groin node status for the survival of T1 and T2 vulval carcinoma patients. Gynecol Oncol. 1995;57:327–34. 7774836 10.1006/gyno.1995.1151

[36] Ho C-M, Chien T-Y, Huang S-H, Wu C-J, Shih B-Y, Chang S-C. Multivariate analysis of the prognostic factors and outcomes in early cervical cancer patients undergoing radical hysterectomy. Gynecol Oncol. 2004;93:458–64. 15099962 10.1016/j.ygyno.2004.01.026

[37] Winer I, Ahmed QF, Mert I, Bandyopadhyay S, Cote M, Munkarah AR, et al. Significance of lymphovascular space invasion in uterine serous carcinoma: what matters more; extent or presence? Int J Gynecol Pathol. 2015;34:47–56. 25473753 10.1097/PGP.0000000000000113

[38] National Comprehensive Cancer Network. (2023). NCCN Clinical Practice Guidelines in Oncology (NCCN Guidelines®): uterine neoplasms (Version 1.2024). https://www.nccn.org/professionals/physician_gls/pdf/uterine.pdf. Accessed Oct 2023 10.6004/jnccn.2023.0006 36791750

[39] Collarino A, Donswijk ML, van Driel WJ, Stokkel MP, Valdés Olmos RA. The use of SPECT/CT for anatomical mapping of lymphatic drainage in vulvar cancer: possible implications for the extent of inguinal lymph node dissection. Eur J Nucl Med Mol Imaging. 2015;42:2064–71. 26219869 10.1007/s00259-015-3127-1

[40] Collarino A, Vidal-Sicart S, Perotti G, Valdés Olmos RA. The sentinel node approach in gynaecological malignancies. Clin Transl Imaging. 2016;4:411–20. 27738629 10.1007/s40336-016-0187-6 PMC5037154

[41] National Comprehensive Cancer Network. (2023). NCCN Clinical Practice Guidelines in Oncology (NCCN Guidelines®): Vulvar Cancer (Version 1.2024). https://www.nccn.org/professionals/physician_gls/pdf/vulvar.pdf. Accessed Oct 2023

[42] Oonk MHM, Planchamp F, Baldwin P, Mahner S, Mirza MR, Fischerová D, et al. European Society of Gynaecological Oncology Guidelines for the Management of Patients with Vulvar Cancer - Update 2023. Int J Gynecol Cancer. 2023;33:1023–43. 37369376 10.1136/ijgc-2023-004486 PMC10359596

[43] Collarino A, Fuoco V, Garganese G, Pasciuto T, de Koster EJ, Florit A, et al. Lymphatic mapping and sentinel node biopsy in vulvar melanoma: the first multicenter study and systematic review. Gynecol Oncol. 2023;170:153–9. 36696819 10.1016/j.ygyno.2023.01.011

[44] Levenback CF, Ali S, Coleman RL, Gold MA, Fowler JM, Judson PL, et al. Lymphatic mapping and sentinel lymph node biopsy in women with squamous cell carcinoma of the vulva: a gynecologic oncology group study. J Clin Oncol. 2012;30:3786–91. 22753905 10.1200/JCO.2011.41.2528 PMC3478573

[45] Van der Zee AGJ, Oonk MH, De Hullu JA, Ansink AC, Vergote I, Verheijen RH, et al. Sentinel node dissection is safe in the treatment of early-stage vulvar cancer. J Clin Oncol. 2008;26:884–9. 18281661 10.1200/JCO.2007.14.0566

[46] Oonk MH, van Hemel BM, Hollema H, de Hullu JA, Ansink AC, Vergote I, et al. Size of sentinel-node metastasis and chances of non-sentinel-node involvement and survival in early stage vulvar cancer: results from GROINSS-V, a multicentre observational study. Lancet Oncol. 2010;11:646–52. 20537946 10.1016/S1470-2045(10)70104-2

[47] Te Grootenhuis NC, van der Zee AGJ, van Doorn HC, van der Velden J, Vergote I, Zanagnolo V, et al. Sentinel nodes in vulvar cancer: Long-term follow-up of the GROningen INternational Study on Sentinel nodes in Vulvar cancer (GROINSS-V) I. Gynecol Oncol. 2016;140:8–14. 26428940 10.1016/j.ygyno.2015.09.077

[48] Garganese G, Collarino A, Fragomeni SM, Rufini V, Perotti G, Gentileschi S, et al. Groin sentinel node biopsy and 18F-FDG PET/CT-supported preoperative lymph node assessment in cN0 patients with vulvar cancer currently unfit for minimally invasive inguinal surgery: The GroSNaPET study. Eur J Surg Oncol. 2017;43:1776–83. 28751058 10.1016/j.ejso.2017.06.018

[49] Collarino A, Garganese G, Valdés Olmos RA, Stefanelli A, Perotti G, Mirk P, et al. Evaluation of dual-timepoint 18F-FDG PET/CT imaging for lymph node staging in vulvar cancer. J Nucl Med. 2017;58:1913–8. 28546331 10.2967/jnumed.117.194332

[50] Koual M, Benoit L, Nguyen-Xuan H-T, Bentivegna E, Azaïs H, Bats A-S. Diagnostic value of indocyanine green fluorescence guided sentinel lymph node biopsy in vulvar cancer: A systematic review. Gynecol Oncol. 2021;161:436–41. 33551201 10.1016/j.ygyno.2021.01.031

[51] Verbeek FPR, Tummers QRJG, Rietbergen DDD, Peters AAW, Schaafsma BE, van de Velde CJH, et al. Sentinel lymph node biopsy in vulvar cancer using combined radioactive and fluorescence guidance. Int J Gynecol Cancer. 2015;25:1086–93. 25768079 10.1097/IGC.0000000000000419 PMC4478233

[52] Deken MM, van Doorn HC, Verver D, Boogerd LSF, de Valk KS, Rietbergen DDD, et al. Near-infrared fluorescence imaging compared to standard sentinel lymph node detection with blue dye in patients with vulvar cancer - a randomized controlled trial. Gynecol Oncol. 2020;159:672–80. 33041071 10.1016/j.ygyno.2020.09.044

[53] Benedetti-Panici P, Maneschi F, Scambia G, Greggi S, Cutillo G, D’Andrea G, et al. Lymphatic spread of cervical cancer: an anatomical and pathological study based on 225 radical hysterectomies with systematic pelvic and aortic lymphadenectomy. Gynecol Oncol. 1996;62:19–24. 8690286 10.1006/gyno.1996.0184

[54] National Comprehensive Cancer Network. (2023). NCCN Clinical Practice Guidelines in Oncology (NCCN Guidelines®): Cervical Cancer (Version 1.2024). https://www.nccn.org/professionals/physician_gls/pdf/cervical.pdf. Accessed Oct 2023

[55] Cibula D, Raspollini MR, Planchamp F, Centeno C, Chargari C, Felix A, et al. ESGO/ESTRO/ESP Guidelines for the management of patients with cervical cancer - Update 2023. Int J Gynecol Cancer. 2023;33:649–66. 37127326 10.1136/ijgc-2023-004429 PMC10176411

[56] Lécuru F, Mathevet P, Querleu D, Leblanc E, Morice P, Daraï E, et al. Bilateral negative sentinel nodes accurately predict absence of lymph node metastasis in early cervical cancer: results of the SENTICOL study. J Clin Oncol. 2011;29:1686–91. 21444878 10.1200/JCO.2010.32.0432

[57] Bats A-S, Buénerd A, Querleu D, Leblanc E, Daraï E, Morice P, et al. Diagnostic value of intraoperative examination of sentinel lymph node in early cervical cancer: a prospective, multicenter study. Gynecol Oncol. 2011;123:230–5. 21893335 10.1016/j.ygyno.2011.08.010

[58] Bats A-S, Mathevet P, Buenerd A, Orliaguet I, Mery E, Zerdoud S, et al. The sentinel node technique detects unexpected drainage pathways and allows nodal ultrastaging in early cervical cancer: insights from the multicenter prospective SENTICOL study. Ann Surg Oncol. 2013;20:413–22. 22911367 10.1245/s10434-012-2597-7

[59] Mathevet P, Lécuru F, Uzan C, Boutitie F, Magaud L, Guyon F, et al. Sentinel lymph node biopsy and morbidity outcomes in early cervical cancer: Results of a multicentre randomised trial (SENTICOL-2). Eur J Cancer. 2021;148:307–15. 33773275 10.1016/j.ejca.2021.02.009

[60] Frumovitz M, Plante M, Lee PS, Sandadi S, Lilja JF, Escobar PF, et al. Near-infrared fluorescence for detection of sentinel lymph nodes in women with cervical and uterine cancers (FILM): a randomised, phase 3, multicentre, non-inferiority trial. Lancet Oncol. 2018;19:1394–403. 30143441 10.1016/S1470-2045(18)30448-0 PMC6580418

[61] Amant F, Mirza MR, Koskas M, Creutzberg CL. Cancer of the corpus uteri. Int J Gynaecol Obstet. 2018;143:37–50. 30306580 10.1002/ijgo.12612

[62] Concin N, Matias-Guiu X, Vergote I, Cibula D, Mirza MR, Marnitz S, et al. ESGO/ESTRO/ESP guidelines for the management of patients with endometrial carcinoma. Int J Gynecol Cancer. 2021;31:12–39. 33397713 10.1136/ijgc-2020-002230

[63] Ballester M, Dubernard G, Lécuru F, Heitz D, Mathevet P, Marret H, et al. Detection rate and diagnostic accuracy of sentinel-node biopsy in early stage endometrial cancer: a prospective multicentre study (SENTI-ENDO). Lancet Oncol. 2011;12:469–76. 21489874 10.1016/S1470-2045(11)70070-5

[64] Cusimano MC, Vicus D, Pulman K, Maganti M, Bernardini MQ, Bouchard-Fortier G, et al. Assessment of sentinel lymph node biopsy vs lymphadenectomy for intermediate- and high-grade endometrial cancer staging. JAMA Surg. 2021;156:157–64. 33175109 10.1001/jamasurg.2020.5060 PMC7658802

[65] McCluggage WG, Wilkinson N. Metastatic neoplasms involving the ovary: a review with an emphasis on morphological and immunohistochemical features. Histopathology. 2005;47:231–47. 16115224 10.1111/j.1365-2559.2005.02194.x

[66] Fournier M, Stoeckle E, Guyon F, Brouste V, Thomas L, MacGrogan G, et al. Lymph node involvement in epithelial ovarian cancer: sites and risk factors in a series of 355 patients. Int J Gynecol Cancer. 2009;19:1307–13. 20009882 10.1111/IGC.0b013e3181b8a07c

[67] Yu H, Wang J, Wu B, Li J, Chen R. Prognostic significance and risk factors for pelvic and para-aortic lymph node metastasis in type I and type II ovarian cancer: a large population-based database analysis. J Ovarian Res. 2023;16:28. 36717897 10.1186/s13048-023-01102-8 PMC9885671

[68] Hassanzadeh M, Hosseini Farahabadi E, Yousefi Z, Kadkhodayan S, Zarifmahmoudi L, Sadeghi R. Lymphatic mapping and sentinel node biopsy in ovarian tumors: a study using intra-operative Tc-99m-Phytate and lymphoscintigraphy imaging. J Ovarian Res. 2016;9:55. 27604260 10.1186/s13048-016-0265-4 PMC5013627

[69] Speth SCJM, Kruitwagen RFPM, Kleppe M, Pooters INA, Van Gorp T, Slangen BFM, et al. Comparison of intraoperative γ-probe imaging and postoperative SPECT/CT in detection of sentinel nodes related to the ovary. J Nucl Med. 2017;58:243–5. 27738006 10.2967/jnumed.116.183426

[70] Nyberg RH, Korkola P, Mäenpää JU. Sentinel node and ovarian tumors: a series of 20 patients. Int J Gynecol Cancer. 2017;27:684–9. 28375928 10.1097/IGC.0000000000000948

[71] Kleppe M, Brans B, Van Gorp T, Slangen BFM, Kruse AJ, Pooters INA, et al. The detection of sentinel nodes in ovarian cancer: a feasibility study. J Nucl Med. 2014;55:1799–804. 25332439 10.2967/jnumed.114.144329

[72] Lago V, Bello P, Montero B, Matute L, Padilla-Iserte P, Lopez S, et al. Clinical application of the sentinel lymph node technique in early ovarian cancer: a pilot study. Int J Gynecol Cancer. 2019;29:377–81. 30718316 10.1136/ijgc-2018-000049

[73] Lago V, Bello P, Montero B, Matute L, Padilla-Iserte P, Lopez S, et al. Sentinel lymph node technique in early-stage ovarian cancer (SENTOV): a phase II clinical trial. Int J Gynecol Cancer. 2020;30:1390–6. 32448808 10.1136/ijgc-2020-001289 PMC7497563

[74] Scambia G, Nero C, Uccella S, Vizza E, Ghezzi F, Cosentino F, et al. Sentinel-node biopsy in early stage ovarian cancer: a prospective multicentre study (SELLY). Int J Gynecol Cancer. 2019;29:1437–9. 31601646 10.1136/ijgc-2019-000886

[75] Zhang RR, Schroeder AB, Grudzinski JJ, Rosenthal EL, Warram JM, Pinchuk AN, et al. Beyond the margins: real-time detection of cancer using targeted fluorophores. Nat Rev Clin Oncol. 2017;14:347–64. 28094261 10.1038/nrclinonc.2016.212 PMC5683405

[76] Kalli KR, Oberg AL, Keeney GL, Christianson TJH, Low PS, Knutson KL, et al. Folate receptor alpha as a tumor target in epithelial ovarian cancer. Gynecol Oncol. 2008;108:619–26. 18222534 10.1016/j.ygyno.2007.11.020 PMC2707764

[77] O’Shannessy DJ, Somers EB, Smale R, Fu Y-S. Expression of folate receptor-α (FRA) in gynecologic malignancies and its relationship to the tumor type. Int J Gynecol Pathol. 2013;32:258–68. 23518909 10.1097/PGP.0b013e3182774562

[78] van Dam GM, Themelis G, Crane LMA, Harlaar NJ, Pleijhuis RG, Kelder W, et al. Intraoperative tumor-specific fluorescence imaging in ovarian cancer by folate receptor-α targeting: first in-human results. Nat Med. 2011;17:1315–9. 21926976 10.1038/nm.2472

[79] Tummers QRJG, Hoogstins CES, Gaarenstroom KN, de Kroon CD, van Poelgeest MIE, Vuyk J, et al. Intraoperative imaging of folate receptor alpha positive ovarian and breast cancer using the tumor specific agent EC17. Oncotarget. 2016;7:32144–55. 27014973 10.18632/oncotarget.8282 PMC5078003

[80] Hoogstins CES, Tummers QRJG, Gaarenstroom KN, de Kroon CD, Trimbos JBMZ, Bosse T, et al. A novel tumor-specific agent for intraoperative near-infrared fluorescence imaging: a translational study in healthy volunteers and patients with ovarian cancer. Clin Cancer Res. 2016;22:2929–38. 27306792 10.1158/1078-0432.CCR-15-2640

[81] Boogerd LSF, Hoogstins CES, Gaarenstroom KN, de Kroon CD, Beltman JJ, Bosse T, et al. Folate receptor-α targeted near-infrared fluorescence imaging in high-risk endometrial cancer patients: a tissue microarray and clinical feasibility study. Oncotarget. 2017;9:791–801. 29416655 10.18632/oncotarget.23155 PMC5787511

[82] Randall LM, Wenham RM, Low PS, Dowdy SC, Tanyi JL. A phase II, multicenter, open-label trial of OTL38 injection for the intra-operative imaging of folate receptor-alpha positive ovarian cancer. Gynecol Oncol. 2019;155:63–8. 31362825 10.1016/j.ygyno.2019.07.010

[83] Tanyi JL, Randall LM, Chambers SK, Butler KA, Winer IS, Langstraat CL, et al. A phase III study of pafolacianine injection (OTL38) for intraoperative imaging of folate receptor-positive ovarian cancer (Study 006). J Clin Oncol. 2023;41:276–84. 36070540 10.1200/JCO.22.00291 PMC12684809

[84] Müller C. Folate-based radiotracers for PET imaging—update and perspectives. Molecules. 2013;18:5005–31. 23629756 10.3390/molecules18055005 PMC6269920

[85] Siegel BA, Dehdashti F, Mutch DG, Podoloff DA, Wendt R, Sutton GP, et al. Evaluation of 111In-DTPA-folate as a receptor-targeted diagnostic agent for ovarian cancer: initial clinical results. J Nucl Med. 2003;44:700–7. 12732670

[86] Fisher RE, Siegel BA, Edell SL, Oyesiku NM, Morgenstern DE, Messmann RA, et al. Exploratory study of 99mTc-EC20 imaging for identifying patients with folate receptor-positive solid tumors. J Nucl Med. 2008;49:899–906. 18483093 10.2967/jnumed.107.049478

[87] Hekman MCH, Boerman OC, Bos DL, Massuger LFAG, Weil S, Grasso L, et al. Improved intraoperative detection of ovarian cancer by folate receptor alpha targeted dual-modality imaging. Mol Pharm. 2017;14:3457–63. 28826214 10.1021/acs.molpharmaceut.7b00464 PMC6150714

[88] Dauphine C, Reicher JJ, Reicher MA, Gondusky C, Khalkhali I, Kim M. A prospective clinical study to evaluate the safety and performance of wireless localization of nonpalpable breast lesions using radiofrequency identification technology. AJR Am J Roentgenol. 2015;204:W720–3. 26001262 10.2214/AJR.14.13201

[89] Constantinidis F, Sakellariou S, Chang SL, Linder S, MacPherson B, Seth S, et al. Wireless localisation of breast lesions with MagSeed. A radiological perspective of 300 cases. Br J Radiol. 2022;95:20211241. 35201906 10.1259/bjr.20211241 PMC10993964

[90] Manca G, Garau LM, Romanini A, Rubello D, Nuzzo A, Barbarello L, et al. Detection of uterine leiomyosarcoma peritoneal lesions by SPECT/CT and ROLL technique. Clin Nucl Med. 2019;44:826–8. 31306202 10.1097/RLU.0000000000002725

[91] Garner HW, Bestic JM, Peterson JJ, Attia S, Wessell DE. Preoperative radioactive seed localization of nonpalpable soft tissue masses: an established localization technique with a new application. Skeletal Radiol. 2017;46:209–16. 27885379 10.1007/s00256-016-2529-x

[92] Hassing CMS, Tvedskov TF, Kroman N, Klausen TL, Drejøe JB, Tvedskov JF, et al. Radioactive seed localisation of non-palpable lymph nodes - A feasibility study. Eur J Surg Oncol. 2018;44:725–30. 29545086 10.1016/j.ejso.2018.02.211

[93] Beneder C, Fuechsel FG, Krause T, Kuhn A, Mueller MD. The role of 3D fusion imaging in sentinel lymphadenectomy for vulvar cancer. Gynecol Oncol. 2008;109:76–80. 18215758 10.1016/j.ygyno.2007.11.045

[94] Valdés Olmos RA, Rietbergen DD, Vidal-Sicart S, Manca G, Giammarile F, Mariani G. Contribution of SPECT/CT imaging to radioguided sentinel lymph node biopsy in breast cancer, melanoma, and other solid cancers: from “open and see” to “see and open.” Q J Nucl Med Mol Imaging. 2014;58:127–39. 24835289

[95] Klapdor R, Länger F, Gratz KF, Hillemanns P, Hertel H. SPECT/CT for SLN dissection in vulvar cancer: Improved SLN detection and dissection by preoperative three-dimensional anatomical localisation. Gynecol Oncol. 2015;138:590–6. 26067332 10.1016/j.ygyno.2015.06.011

[96] Navarro A-S, Angeles MA, Migliorelli F, Illac C, Martínez-Gómez C, Leray H, et al. Comparison of SPECT-CT with intraoperative mapping in cervical and uterine malignancies. Int J Gynecol Cancer. 2021;31:679–85. 33649157 10.1136/ijgc-2020-002198

[97] Hoogendam JP, Veldhuis WB, Hobbelink MGG, Verheijen RHM, van den Bosch MAAJ, Zweemer RP. 99mTc SPECT/CT versus planar lymphoscintigraphy for preoperative sentinel lymph node detection in cervical cancer: a systematic review and metaanalysis. J Nucl Med. 2015;56:675–80. 25858041 10.2967/jnumed.114.152439

[98] Martínez A, Zerdoud S, Mery E, Bouissou E, Ferron G, Querleu D. Hybrid imaging by SPECT/CT for sentinel lymph node detection in patients with cancer of the uterine cervix. Gynecol Oncol. 2010;119:431–5. 20822803 10.1016/j.ygyno.2010.08.001

[99] Pandit-Taskar N, Gemignani ML, Lyall A, Larson SM, Barakat RR, Abu Rustum NR. Single photon emission computed tomography SPECT-CT improves sentinel node detection and localization in cervical and uterine malignancy. Gynecol Oncol. 2010;117:59–64. 20117827 10.1016/j.ygyno.2009.12.021

[100] Buda A, Elisei F, Arosio M, Dolci C, Signorelli M, Perego P, et al. Integration of hybrid single-photon emission computed tomography/computed tomography in the preoperative assessment of sentinel node in patients with cervical and endometrial cancer: our experience and literature review. Int J Gynecol Cancer. 2012;22:830–5. 22617479 10.1097/IGC.0b013e318253496f

[101] Garganese G, Bove S, Zagaria L, Moro F, Fragomeni SM, Ieria FP, et al. Fusion of ultrasound and 3D single-photon-emission computed tomography/computed tomography to identify sentinel lymph nodes in vulvar cancer: feasibility study. Ultrasound Obstet Gynecol. 2019;54:545–51. 31152573 10.1002/uog.20364

[102] Narayanan P, Sahdev A. The role of 18F-FDG PET CT in common gynaecological malignancies. Br J Radiol. 2017;90:20170283. 28830238 10.1259/bjr.20170283 PMC5963379

[103] Crivellaro C, Signorelli M, Guerra L, De Ponti E, Buda A, Dolci C, et al. 18F-FDG PET/CT can predict nodal metastases but not recurrence in early stage uterine cervical cancer. Gynecol Oncol. 2012;127:131–5. 22772064 10.1016/j.ygyno.2012.06.041

[104] Crivellaro C, Signorelli M, Guerra L, De Ponti E, Pirovano C, Fruscio R, et al. Tailoring systematic lymphadenectomy in high-risk clinical early stage endometrial cancer: the role of 18F-FDG PET/CT. Gynecol Oncol. 2013;130:306–11. 23707673 10.1016/j.ygyno.2013.05.011

[105] Aide N, Markovina S, Ferrero A. Is it time to include [18F]FDG-PET/CT in the diagnostic work-up for lymph node staging in cN0 vulvar cancer patients? Eur J Nucl Med Mol Imaging. 2021;48:3043–5. 33768280 10.1007/s00259-021-05317-z

[106] Triumbari EKA, de Koster EJ, Rufini V, Fragomeni SM, Garganese G, Collarino A. 18F-FDG PET and 18F-FDG PET/CT in vulvar cancer: a systematic review and meta-analysis. Clin Nucl Med. 2021;46:125–32. 33234921 10.1097/RLU.0000000000003411

[107] Rufini V, Garganese G, Ieria FP, Pasciuto T, Fragomeni SM, Gui B, et al. Diagnostic performance of preoperative [18F]FDG-PET/CT for lymph node staging in vulvar cancer: a large single-centre study. Eur J Nucl Med Mol Imaging. 2021;48:3303–14. 33619601 10.1007/s00259-021-05257-8 PMC8426310

[108] Dendl K, Koerber SA, Finck R, Mokoala KMG, Staudinger F, Schillings L, et al. 68Ga-FAPI-PET/CT in patients with various gynecological malignancies. Eur J Nucl Med Mol Imaging. 2021;48:4089–100. 34050777 10.1007/s00259-021-05378-0 PMC8484099

[109] Chen J, Xu K, Li C, Tian Y, Li L, Wen B, et al. [68Ga]Ga-FAPI-04 PET/CT in the evaluation of epithelial ovarian cancer: comparison with [18F]F-FDG PET/CT. Eur J Nucl Med Mol Imaging. 2023;50:4064–76. 37526694 10.1007/s00259-023-06369-z

[110] Decesare SL, Fiorica JV, Roberts WS, Reintgen D, Arango H, Hoffman MS, et al. A pilot study utilizing intraoperative lymphoscintigraphy for identification of the sentinel lymph nodes in vulvar cancer. Gynecol Oncol. 1997;66:425–8. 9299256 10.1006/gyno.1997.4798

[111] de Hullu JA, Doting E, Piers DA, Hollema H, Aalders JG, Koops HS, et al. Sentinel lymph node identification with technetium-99m-labeled nanocolloid in squamous cell cancer of the vulva. J Nucl Med. 1998;39:1381–5. 9708512

[112] Schneebaum S, Even-Sapir E, Cohen M, Shacham-Lehrman H, Gat A, Brazovsky E, et al. Clinical applications of gamma-detection probes - radioguided surgery. Eur J Nucl Med. 1999;26:S26-35. 10199930 10.1007/pl00014792

[113] Holman LL, Levenback CF, Frumovitz M. Sentinel lymph node evaluation in women with cervical cancer. J Minim Invasive Gynecol. 2014;21:540–5. 24407177 10.1016/j.jmig.2013.12.095 PMC4283488

[114] Povoski SP, Hall NC, Murrey DA, Chow AZ, Gaglani JR, Bahnson EE, et al. Multimodal imaging and detection approach to 18F-FDG-directed surgery for patients with known or suspected malignancies: a comprehensive description of the specific methodology utilized in a single-institution cumulative retrospective experience. World J Surg Oncol. 2011;9:152. 22112047 10.1186/1477-7819-9-152 PMC3247132

[115] Yen T-C, See L-C, Lai C-H, Yah-Huei CW, Ng K-K, Ma S-Y, et al. 18F-FDG uptake in squamous cell carcinoma of the cervix is correlated with glucose transporter 1 expression. J Nucl Med. 2004;45:22–9. 14734665

[116] Collarino A, Florit A, Bizzarri N, Lanni V, Morganti S, De Summa M, et al. Radioguided surgery with β decay: a feasibility study in cervical cancer. Phys Med. 2023;113: 102658. 37603908 10.1016/j.ejmp.2023.102658

[117] Tiourina T, Arends B, Huysmans D, Rutten H, Lemaire B, Muller S. Evaluation of surgical gamma probes for radioguided sentinel node localisation. Eur J Nucl Med. 1998;25:1224–31. 9724369 10.1007/s002590050288

[118] Dell’Oglio P, Meershoek P, Maurer T, Wit EMK, van Leeuwen PJ, van der Poel HG, et al. A DROP-IN gamma probe for robot-assisted radioguided surgery of lymph nodes during radical prostatectomy. Eur Urol. 2021;79:124–32. 33203549 10.1016/j.eururo.2020.10.031

[119] Baeten IGT, Hoogendam JP, Braat AJAT, Zweemer RP, Gerestein CG. Feasibility of a drop-in γ-probe for radioguided sentinel lymph detection in early-stage cervical cancer. EJNMMI Res. 2022;12:36. 35723832 10.1186/s13550-022-00907-w PMC9209631

[120] Fuerst B, Sprung J, Pinto F, Frisch B, Wendler T, Simon H, et al. First robotic SPECT for minimally invasive sentinel lymph node mapping. IEEE Trans Med Imaging. 2016;35:830–8. 26561283 10.1109/TMI.2015.2498125

[121] Azargoshasb S, Houwing KHM, Roos PR, van Leeuwen SI, Boonekamp M, Mazzone E, et al. Optical navigation of the drop-in γ-probe as a means to strengthen the connection between robot-assisted and radioguided surgery. J Nucl Med. 2021;62:1314–7. 33419942 10.2967/jnumed.120.259796 PMC8882900

[122] Azargoshasb S, van Alphen S, Slof LJ, Rosiello G, Puliatti S, van Leeuwen SI, et al. The Click-On gamma probe, a second-generation tethered robotic gamma probe that improves dexterity and surgical decision-making. Eur J Nucl Med Mol Imaging. 2021;48:4142–51. 34031721 10.1007/s00259-021-05387-z PMC8566398

[123] Vidal-Sicart S, Seva A, Campos F, Sánchez N, Alonso I, Pahisa J, et al. Clinical use of an opto-nuclear probe for hybrid sentinel node biopsy guidance: first results. Int J Comput Assist Radiol Surg. 2019;14:409–16. 29968113 10.1007/s11548-018-1816-5

[124] Ookubo T, Inoue Y, Kim D, Ohsaki H, Mashiko Y, Kusakabe M, et al. Characteristics of magnetic probes for identifying sentinel lymph nodes. Annu Int Conf IEEE Eng Med Biol Soc. 2013;2013:5485–8. 24110978 10.1109/EMBC.2013.6610791

[125] Douek M, Klaase J, Monypenny I, Kothari A, Zechmeister K, Brown D, et al. Sentinel node biopsy using a magnetic tracer versus standard technique: the SentiMAG Multicentre Trial. Ann Surg Oncol. 2014;21:1237–45. 24322530 10.1245/s10434-013-3379-6

[126] van den Berg NS, Simon H, Kleinjan GH, Engelen T, Bunschoten A, Welling MM, et al. First-in-human evaluation of a hybrid modality that allows combined radio- and (near-infrared) fluorescence tracing during surgery. Eur J Nucl Med Mol Imaging. 2015;42:1639–47. 26109329 10.1007/s00259-015-3109-3

[127] Olmos RAV, Vidal-Sicart S, Nieweg OE. SPECT-CT and real-time intraoperative imaging: new tools for sentinel node localization and radioguided surgery? Eur J Nucl Med Mol Imaging. 2009;36:1–5. 18931842 10.1007/s00259-008-0955-2

[128] Vidal-Sicart S, Vermeeren L, Solà O, Paredes P, Valdés-Olmos RA. The use of a portable gamma camera for preoperative lymphatic mapping: a comparison with a conventional gamma camera. Eur J Nucl Med Mol Imaging. 2011;38:636–41. 21174091 10.1007/s00259-010-1682-z

[129] Heller S, Zanzonico P. Nuclear probes and intraoperative gamma cameras. Semin Nucl Med. 2011;41:166–81. 21440694 10.1053/j.semnuclmed.2010.12.004

[130] Markus A, Ray ASC, Bolla D, Müller J, Diener P-A, Wendler T, et al. Sentinel lymph node biopsy in endometrial and cervical cancers using freehand SPECT—first experiences. Gynecol Surg. 2016;13:499–506.

[131] Bluemel C, Safak G, Cramer A, Wöckel A, Gesierich A, Hartmann E, et al. Fusion of freehand SPECT and ultrasound: First experience in preoperative localization of sentinel lymph nodes. Eur J Nucl Med Mol Imaging. 2016;43:2304–12. 27311920 10.1007/s00259-016-3443-0

[132] Azargoshasb S, Molenaar L, Rosiello G, Buckle T, van Willigen DM, van de Loosdrecht MM, et al. Advancing intraoperative magnetic tracing using 3D freehand magnetic particle imaging. Int J Comput Assist Radiol Surg. 2022;17:211–8. 34333740 10.1007/s11548-021-02458-2 PMC8738628

[133] Wendler T, Traub J, Ziegler SI, Navab N. Navigated three dimensional beta probe for optimal cancer resection. Med Image Comput Comput Assist Interv. 2006;9:561–9. 17354935 10.1007/11866565_69

[134] Togami S, Fukuda M, Mizuno M, Yanazume S, Kobayashi H. Efficacy and prognosis of robotic surgery with sentinel node navigation surgery in endometrial cancer. J Gynecol Oncol. 2023;34: e68. 37293801 10.3802/jgo.2023.34.e68 PMC10627747

[135] Mohammad A, Hunter MI. Robot-assisted sentinel lymph node mapping and inguinal lymph node dissection using near-infrared dluorescence in vulvar cancer. J Minim Invasive Gynecol. 2019;26:968–72. 30959199 10.1016/j.jmig.2019.04.002

[136] Baeten IGT, Hoogendam JP, Braat AJAT, de Keizer B, Gerestein CG, Zweemer RP. Learning curve and factors influencing successful robot-assisted bilateral sentinel lymph node mapping in early-stage cervical cancer: an observational cohort study. Expert Rev Med Devices. 2023;20:589–96. 37278067 10.1080/17434440.2023.2212157

[137] Andras I, Mazzone E, van Leeuwen FWB, De Naeyer G, van Oosterom MN, Beato S, et al. Artificial intelligence and robotics: a combination that is changing the operating room. World J Urol. 2020;38:2359–66. 31776737 10.1007/s00345-019-03037-6

[138] Bourdel N, Collins T, Pizarro D, Debize C, Grémeau A-S, Bartoli A, et al. Use of augmented reality in laparoscopic gynecology to visualize myomas. Fertil Steril. 2017;107:737–9. 28089570 10.1016/j.fertnstert.2016.12.016

[139] Lecointre L, Verde J, Hubele F, Salvadori J, Goffin L, Akladios C, et al. Preoperative SPECT/CT + intraoperative CT fusion enabling surgical augmented reality to target sentinel lymph node in endometrial cancer. EJNMMI Phys. 2022;9:81. 36414716 10.1186/s40658-022-00506-7 PMC9681940

